# Water Transfer Between Bamboo Culms in the Period of Sprouting

**DOI:** 10.3389/fpls.2019.00786

**Published:** 2019-06-12

**Authors:** Dongming Fang, Tingting Mei, Alexander Röll, Dirk Hölscher

**Affiliations:** ^1^State Key Laboratory of Subtropical Silviculture, Zhejiang A&F University, Lin’an, China; ^2^Tropical Silviculture and Forest Ecology, University of Göttingen, Göttingen, Germany

**Keywords:** bamboo shoots, nighttime flux, rhizome, sap flux, water exchange

## Abstract

Bamboo culms are connected to neighboring culms via rhizomes, which enable resource exchange between culms. We assessed water transfer between established and neighboring, freshly sprouted culms by thermal dissipation probes (TDP) inserted into culms and the connecting rhizome. During the early phase of sprouting, highest sap flux densities in freshly sprouted culms were observed at night, whereas neighboring established culms had high sap flux densities during daytime. After leaf flushing on freshly sprouted culms, the nighttime peaks disappeared and culms switched to the diurnal sap flux patterns with daytime maxima as observed in established culms. TDP in rhizomes indicated water flowing from the established to the freshly sprouted culms. When the established culms of a clump were cut, freshly sprouted culms without leaves reduced sap flux densities rates by 79%. Our findings thus suggest that bamboos exchange water via rhizomes and that nighttime fluxes are highly important for the support of freshly sprouted culms. The (water) resource support may facilitate the very fast growth of the bamboo shoots, and enable the colonizing of new places.

## Introduction

Plants with connected roots or rhizomes have the possibility to share resources with each other directly (Baret and DesRochers, 2011). Resource exchange among connected individuals, referred to as “physiological integration” (Lau and Young, 1988; Caraco and Kelly, 1991; Kroon et al., 1996), has been intensively studied and seems to be relatively widespread in herbaceous species (Alpert and Mooney, 1986; Lau and Young, 1988; Chapman et al., 1992; Kroon et al., 1996; Stueffer et al., 1996). In tree species such as lodgepole pine (Fraser et al., 2006), aspen (Baret and DesRochers, 2011), and poplar (Adonsou et al., 2016) resource exchange via connected roots was also observed. The direction of transfer and the transferred amounts depended on the status of the connected plants; e.g., plants that suffered drought received water from watered neighbors, in which the amount of transferred water was related to the leaf water potential or leaf area of the connected plants (Kroon et al., 1996; Adonsou et al., 2016). Such resource integration was shown to be of critical importance for new ramets grown from the parental root systems of aspen (*Populus tremuloides*) (Baret and DesRochers, 2011).

As rhizomatous monocot species, bamboos are well known for their fast expansion via their underground rhizome system as well as the rapid growth of freshly sprouted culms (Liese and Köhl, 2015). After emerging from the soil, bamboo culms can attain their full heights within 1 or 2 months, with maximum growth rates up to 10–80 cm per day (Liese and Köhl, 2015; Song et al., 2016). Some species may even grow up to 1 m per day during the fast growing phase (Ueda, 1960). This leads to the question of where developing culms, with no leaves and only few roots, get the resources to sustain such growth rates. Important mechanisms include nutrient storage in the rhizome as well as resource translocation from connected mature culms (Li et al., 1998a,b; Liese and Köhl, 2015). A study on Moso bamboo (*Phyllostachys pubescens*) revealed that the content of non-structural carbohydrates in mature culms declined substantially during the “explosive growth” period of neighboring, young bamboo shoots due to the translocation of carbohydrates from mature to young culms via the underground rhizomes (Song et al., 2016). Applying deuterium tracing on culms in a clump of *Bambusa blumeana*, Dierick et al. (2010) found higher deuterium concentration than the background values in neighboring culms close to the labeled culms. This elevated deuterium concentration was thought to imply water transfer among the culms via the rhizomes. In another study on Moso bamboo all rhizomes of several culms were cut and the culms thus disconnected from the clump’s rhizome network. The culms with cut rhizomes subsequently consumed 20% less water than neighboring culms with intact rhizomes (Zhao et al., 2016).

Our study was implemented on three clumpy bamboo species during the phase of vegetative sprouting of fresh culms. The aim was to assess water transfer among established culms and sprouting culms with thermal dissipation probes (TDP) and a cutting experiment.

## Materials and Methods

### Study Site and Bamboos

The study was conducted in a bamboo garden in Bogor, Indonesia (6°33′40″ S, 106°43′27″ E, 182 m asl). Rainfall in Bogor is 3978 mm per year and the mean annual temperature is 25.6°C (Van Den Besselaar et al., 2014). The months between October and May are particularly wet. During this wet period, new shoots of bamboos sprout from the soil and grow to their full height. This study was conducted in clumps of *B. vulgaris* and *Gigantochloa apus*; culm heights were 17.9 ± 0.8 m (mean ± SD) and 16.2 ± 2.7 m and culm diameters at breast height were 7.0 ± 0.4 cm and 7.9 ± 1.1 cm, respectively. In the clumps, established culms and freshly sprouted culms were connected via rhizomes of 30 to 50 cm length. Unlike the hollow culms of the bamboos, the rhizomes are solid. During the nearly 4-month experimental period from December 2012 to March 2013, fresh culms sprouted and grew to their full height. Culm growth was slowed down during the period of leaf development.

### Sap Flux in Culms and the Rhizome, and Measurements of Radiation

**FIGURE 1:**
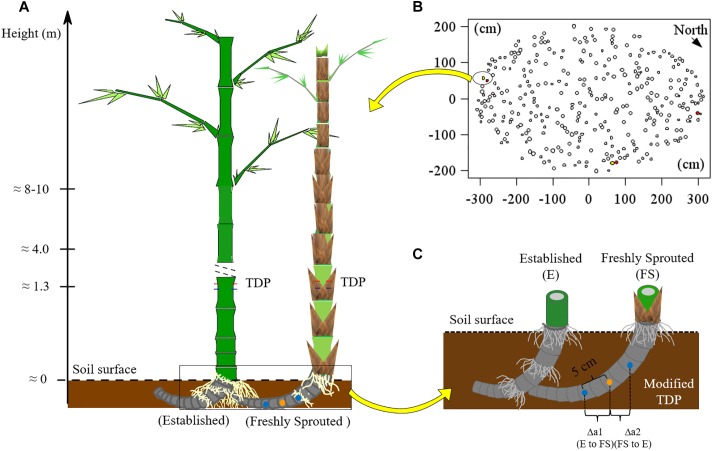
**(A)** Experimental scheme for applying TDP methods; **(B)** the locations of monitored culms in a clump of *B. vulgaris* (*n* = 3 pairs of culms). The red, yellow and white filled circles represented the labeled established, the neighboring freshly sprouted and the other culms, respectively. **(C)** Setup of self-made modified thermal dissipation probes (TDP) with two reference probes installed at equal distance from the central heating probe on the rhizome of *B. vulgaris*. The orange point represents the heating probe, the blue points are the unheated reference probes. Photos showing the installation of TDP on bamboo culms and of modified TDP on bamboo rhizomes are provided in Appendix Figure S1.

For *B. vulgaris*, three pairs of established and attached freshly sprouted culms were selected at the culm edge (Figure 1A,B). The connection between established and freshly sprouted culms was verified by partially removing the topsoil and directly observing the rhizomes. Sap flux densities (*J*_s_, g cm^−2^ h^−1^) in bamboo culms were monitored with TDP (Granier, 1985). Each TDP consists of two probes – a heated probe and a reference one, and a thermal couple is built into each probe to detect thermal dynamic. The temperature around the heated probe is negatively correlated with *J*_s_. The temperature differential between heated and reference probe were used for monitoring and calculating *J*_s_ (Lu et al., 2004). On each studied bamboo culm, three pairs of TDP with 1 cm sensor length were inserted into the culm walls at breast height (Figure 1A and Appendix Figure S1A). The heated probe, powered by 120 mA, was installed 10 cm above the reference probe. Voltage signals were recorded every 30 s and averaged every 10 min (CR1000 data loggers and AM16/32 multiplexer, Campbell Scientific Inc., United States). *J*_s_ of established culms was first calculated based on Granier’s equation (Granier, 1985), and *J*_s_ was further corrected by multiplying it with species-specific calibration parameters (*SSCP*; 2.79 for *B. vulgaris*; 3.32 for *G. apus*; Mei et al., 2016).

$$J_{s}=119\times {\left(\frac{\Delta T_{\max}-\Delta T}{\Delta T}\right)}^{1.231}\times 0.36\times \mathrm{SSCP}$$

Where Δ*T* is the temperature difference between the heated and reference probe and Δ*T_max_* is the maximal Δ*T* within a given day; Δ*T_max_* usually occurs during the night and is used to set zero-flux conditions (*J*_s_ = 0). The model parameters 119 and 1.231 were empirically derived by Granier (1985). These original parameters were found to substantially underestimate *J*_s_ in the bamboo species *B. vulgaris* and *G. apus* (Mei et al., 2016), which is why the mentioned *SSCP* were applied.

To measure *J*_s_ and detect directions of sap flow in the rhizome between established and freshly sprouted culms, we built self-made, modified TDP sensors with three probes instead of two. The modified TDP consisted of one central heating and two unheated reference probes. The heating probe was installed at the mid-point of the rhizome between the culms, and the two reference probes were installed at 5 cm distance from the heated probe, one on each side (Figure 1C and Appendix Figure S1B). The temperature differences between each reference probe and the heating probe were recorded, stored and used to calculate rhizome *J*_s_ in the same way as described above for the “standard” TDP sensors. The directions of sap flow in rhizome were determined by comparing the two derived voltage signals, and the reference probe with the lower signal value was assigned the downstream position of sap flow. This was based on the assumption that sap flow brought the heat energy to the downstream sensor and that this heat would increase the temperature of the downstream sensor and thus a smaller temperature difference between the heating and the downstream reference probe than that between the heating and the upstream reference probe. In this case, the signal value from the downstream sensor was smaller than that from the upstream sensor. To test this assumption, we simulated the heat field around the heating and the two reference sensors under different sap flow densities with the ANSYS model (CFX 17.0, ANSYS Inc., Pennsylvania, United States; Mei et al., 2017), and the simulated results confirmed our initial assumption (Appendix Figures S2–S4).

Within 600 meters of the *B. vulgaris* site, we set up a micrometeorological station. A pyranometer (CS300, Campbell Scientific Inc., United States) was installed to measure radiation. The data was stored on a CR1000 data logger with the same recording intervals as described for the TDP measurements.

### Cutting Experiment

Additionally to directly monitoring the water transfer via rhizome with modified TDP, a cutting experiment was implemented to explore the influence of established culms on the water status of freshly sprouted culms. Two clumps of *G. apus* in a bamboo garden were selected, one for the cutting experiment and the other as a control. Both clumps had a similar density of culms (∼ 18 culms m^−2^). All established culms in the cutting experiment clump were removed, so that only freshly sprouted culms remained. In the control clump, no culms were cut.

From 29 Dec 2012 to 10 Feb 2013, *J*_s_ of five freshly sprouted culms in the cutting experiment clump was monitored by TDP as described in section 2.2. The culms had a height of around 2 m at the beginning of the experiment and had no leaves. The monitoring stopped after about 40 days, when the culms were collapsing. Until then, the culms had reached approx. 5 – 8 m in height without any leaf development. In the control clump, we monitored *J*_s_ of five established culms but not freshly sprouted ones. To estimate the *J*_s_ of freshly sprouted culms in the *G. apus* control clump, we used an indirect assessment involving a nearby long-term monitoring *B. vulgaris* clump. There, we measured *J*_s_ of both established and freshly sprouted culms. We found a significant linear relationship (slope = 0.63, with no intercept) between *J*_s_ of established culms in the *B. vulgaris* and *G. apus* control clumps (*R*^2^ = 0.9; *P* < 0.01; Appendix Figure S5). We assume that the freshly sprouted culms of the two species have the same relationship as established culms and estimated *J*_s_ of freshly sprouted culms in the control *G. apus* clump by multiplying the observed values of freshly sprouted *B. vulgaris* by 0.63 (Appendix Figure S5).

### Statistics

**FIGURE 2:**
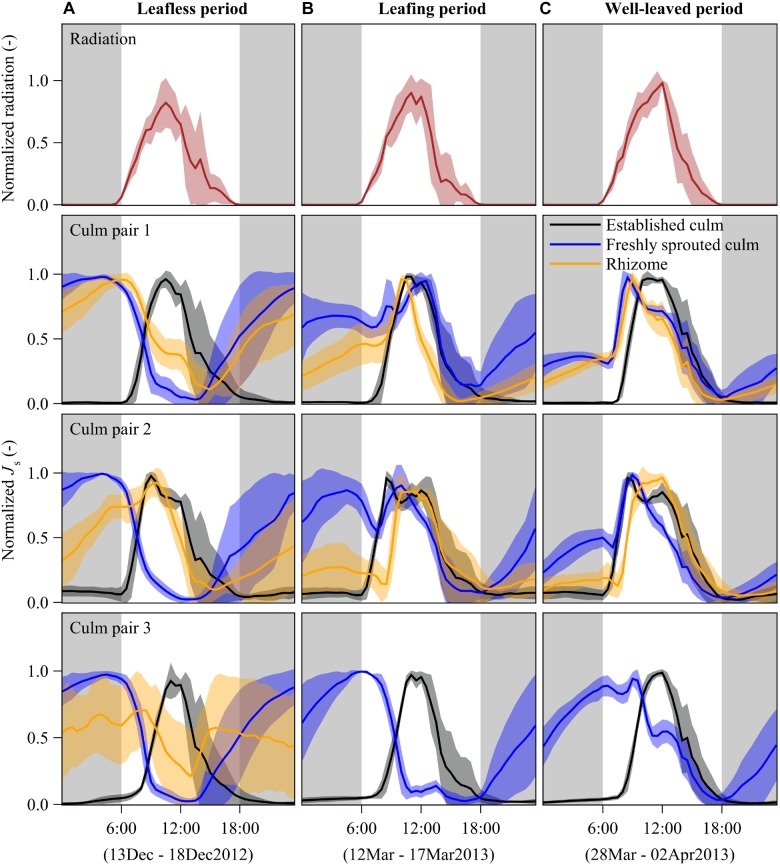
Diurnal patterns of half-hourly radiation (first row) and sap flux densities (*J*_s_) of *B. vulgaris* (rows 2 – 4) during leafless **(A)**, leafing **(B)**, and well-leaved period **(C)** of freshly sprouted culms. The presented lines are means of 6 days of measurements for each of the three periods; the error corridors around the lines indicate standard deviations. For each freshly sprouted culm, *J*_s_ is also displayed for the neighboring established culm and the connecting rhizome. Gray background indicates nighttime (18:00 to 6:00). Due to sensor malfunctioning, rhizome data for culm pair three is missing and thus not shown in panels **B,C**.

**FIGURE 3:**
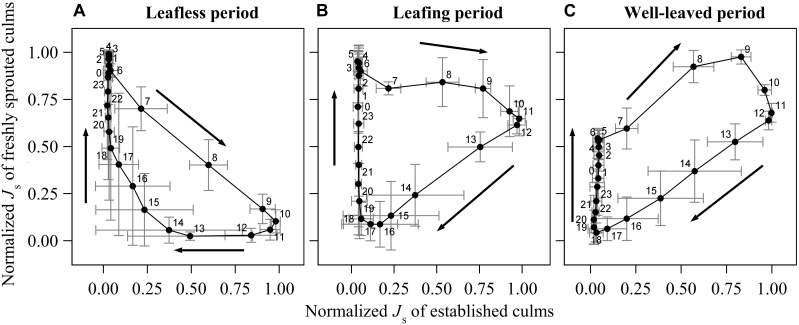
Diurnal change of normalized sap flux density (*J*_s_) of the freshly sprouted culms to the normalized *J*_s_ of established culms in the leafless **(A)**, leafing **(B)** and well-leaved period **(C)** of the freshly sprouted culms of *B. vulgaris*. In each sub-figure, data was averaged from six consecutive sunny days of three culm pairs. The *X*- and *Y*- error bars on each dot are standard deviations of the respective 6 days.

To compare the patterns of *J*_s_ among the rhizome, established and freshly sprouted culms, we normalized the half-hourly *J*_s_ of each day (by fitting them to a range from 0 to 1) and plotted average hourly values of six sunny days (Figure 2). To further explore the relative change between established and freshly sprouted culms, we further plotted the normalized *J*_s_ values of newly sprouted culms directly versus the normalized *J*_s_ of established culms (Figure 3).

**FIGURE 4:**
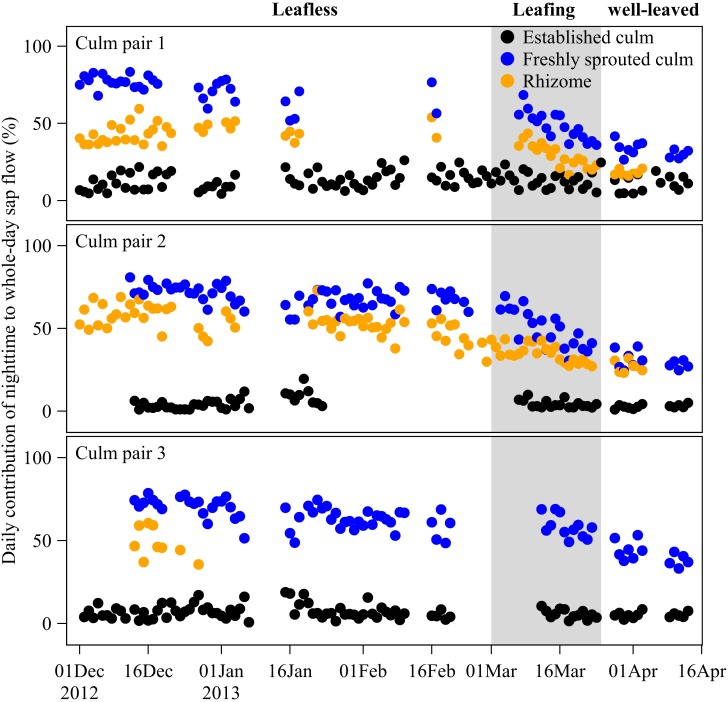
Daily contribution (%) of nighttime to whole-day sap flow in freshly sprouted culms, established culms and the connecting rhizomes of three culm pairs of *B. vulgaris* in the first months after sprouting. The gray area indicates the leafing period of freshly sprouted culms, i.e., the transition phase from a leafless to a well-leaved state.

To explore the contribution of nighttime to whole-day sap flow, we calculated the ratio between nighttime (18:00 to 6:00 in next morning) and whole-day accumulated sap flow (24 h) of established and freshly sprouted culms and of the rhizomes connecting them (Figure 4).

**FIGURE 5:**
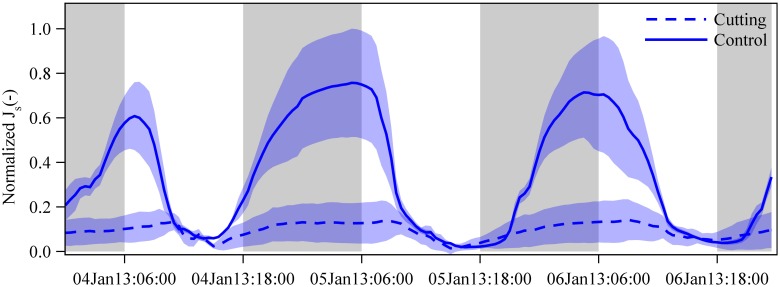
Normalized sap flux densities (*J*_s_) in freshly sprouted culms of *G. apus* in cutting (dashed lines) and control clumps (solid line). In the cutting experiment clump, all established culms were removed, leaving only freshly sprouted culms. Due to missing measured data, we estimated *J*_s_ of freshly sprouted *G. apus* culms in the control clump by multiplying *J*_s_ of freshly sprouted *B. vulgaris* culms in a nearby clump by a ratio of 0.63 (see section “Materials and Methods” and Appendix Figure S5). Averaged data from 3 and 5 culms for the control and cutting clump, respectively, normalized by setting the hourly maximum and minimum of both datasets to one and zero. Lines represent means and error corridors around the lines indicate standard deviations.

To compare the patterns of *J*_s_ between the cutting and control freshly sprouted culms in the cutting experiment, we averaged half-hourly *J*_s_ of 5 culms in each clump, and normalized the pooled data of both clumps by selecting one common hourly maximum (set to one) and minimum (set to zero) within three consecutive days of measurements (Figure 5).

All data analyses and plotting were performed with SAS 9.4 (SAS Institute Inc., Cary, NC, United States).

## Results

### Diurnal Patterns of Sap Flux Densities in Culms and Rhizome

In established culms, sap flux density (*J*_s_) showed a typical diurnal pattern corresponding approximately to the diurnal patterns of radiation. With rising radiation in the morning, *J*_s_ started to increase until reaching its peak values at around midday, gradually decreased in the afternoon and remained close to zero during the night (Figure 2).

In freshly sprouted culms, *J*_s_ switched from a day-night reversal mode through a transition mode to a similar diurnal pattern as that of established culms over the leafless, leafing, and well-leaved periods, respectively. In the leafless period, nighttime sap flux dominated. *J*_s_ increased from near zero at sunset time to its peak around sunrise. The nighttime-dominated pattern switched gradually to the normal daytime-dominated pattern after leaves developed. In the well-leaved period, 3 months after sprouting, maximum *J*_s_ was higher during the daytime than during the nighttime (Figure 2).

In rhizomes, there was substantial *J*_s_ at night during times of high *J*_s_ in freshly sprouted culms in the leafless period. During the daytime, the *J*_s_ in rhizomes differed among the three culm pairs (Figure 2A), which may be attributed to different culm elongation stages. Once the leaves of young culms were well established (Figure 2B), *J*_s_ patterns were more synchronized in rhizomes, established and young bamboo culms, usually with peaks between 9 am and midday. Based on observed temperature differences as measured with modified TDPs (Appendix Figure S2) and results from simulations (Appendix Figures S3, S4), we infer that the net sap flow in rhizomes was mainly from the established culms to the freshly sprouted culms in both the leafless and the well-leaved observation period. However, we cannot exclude the possibility of coexisting bidirectional sap flow in rhizomes.

### Comparison Between Freshly Sprouted Culms and Established Culms

During the leafless period of freshly sprouted culms, they had near maximum rates of normalized hourly sap flux (*J*_s_max_) from about 10 pm to 6 am, when *J*_s_ of established culms was near its minimum (<10% of *J*_s_max_) (Figure 3A). Sap flux in leafless young culms sharply decreased as soon as sap flux in established culms increased in the morning. *J*_s_max_ in established culms coincided with near minimum values in freshly sprouted culms (9 am to 12 pm). *J*_s_ in freshly sprouted culms increased back to 25% of its daily maximum as soon as *J*_s_ in established culms dropped below 20% of *J*_s_max_ (around 4 pm).

In freshly sprouted culms in the transition period (leafing), *J*_s_max_ was also observed in the early morning hours, but the subsequent decline in *J*_s_ was much slower; *J*_s_ was still around 70% of *J*_s_max_ at noon, when *J*_s_ in established culms was at its highest (Figure 3B). Over the afternoon hours (12 to 6 pm), *J*_s_ in leafing young culms declined to near zero. It subsequently gradually increased until the early morning hour maximum; during this time, *J*_s_ of established culms remained below 10% of *J*_s_max_.

In well-leaved freshly sprouted culms, substantial *J*_s_ as well as *J*_s_max_ were observed between 5 am and noon, thus coinciding with *J*_s_max_ in established culms. In the afternoon, *J*_s_ in the young culms gradually drops to near minimum (around 6 pm). However, in contrast to established culms (with marginal *J*_s_ between 6 pm and 6 am), *J*_s_ in well-leaved freshly sprouted culms starts to slowly and consistently rise again after sunset, reaching about 60% of *J*_s_max_ until sunrise (Figure 3C).

### Contribution of Nighttime to Whole-Day Sap Flow

Over the leafless, leafing, and well-leaved periods of newly sprouted culms, nighttime accumulated *J*_s_ of neighboring established culms remained at 8 ± 1% of whole-day accumulated *J*_s_ (Figure 4). In contrast, the contribution of nighttime to whole-day accumulated *J*_s_ of freshly sprouted culms decreased from 69 ± 3% to 52 ± 6% to 35 ± 6% over the three periods (Figure 4). Nighttime accumulated *J*_s_ in rhizomes accounted for 49 ± 5%, 32 ± 4%, and 23 ± 6% of whole-day accumulated *J*_s_ over the three periods (Figure 4). Even though the nighttime contributions of rhizomes were thus consistently smaller than for freshly sprouted culms, they showed a similar decreasing trend over the three periods (Figure 4).

### Influence of Cutting Established Culms on Freshly Sprouted Culms

After removing all established culms in a *G. apus* clump, daily sap flux of freshly sprouted leafless culms was estimated to be 79% lower than the corresponding values of a *G. apus* clump where established culms had not been removed (Figure 5); the latter was assessed indirectly by applying the ratio of 0.63 between *J*_s_ of *G. apus* and *B. vulgaris* (Appendix Figure S5). Taking into account uncertainties in the ratio (range: 0.55 to 0.84), the corresponding relative difference of *J*_s_ between cutting and control clump is between 76 and 84%.

## Discussion

It is generally accepted that micrometeorological factors such as solar radiation and vapor pressure deficit are the main drivers of day-to-day fluctuations in tree water use and that limited soil water availability can constrain tree water use (O’brien et al., 2004; Kume et al., 2007). However, water use patterns may vary under special circumstances, e.g., in freshly sprouted leafless bamboo culms. In our study, we found that diurnal *J*_s_ patterns in freshly sprouted bamboo culms changed from the leafless to the well-leaved period (Figure 2, 3). During the leafless period, diurnal *J*_s_ patterns in freshly sprouted bamboo culms typically differed from patterns in neighboring established culms, i.e., freshly sprouted culms had high sap flux during the night and low sap flux during the daytime. The nighttime maxima imply that the *J*_s_ pattern of freshly sprouted bamboo culms are not always controlled by the classic micrometeorological drivers, particularly during the early growing stages when culms are still without leaves and branches. The neighboring established culms most likely play an important supportive role for the freshly sprouted culms (Fraser et al., 2006; Liese and Köhl, 2015). As such, when all the established culms in a *G. apus* clump in our study were cut, *J*_s_ in neighboring, newly sprouted culms was reduced by 79% (Figure 5).

Tentatively applying the available adjusted *J*_s_ formula as specified for established bamboo culms (Mei et al., 2016) to the rhizomes and freshly sprouted culms in our study suggests that during the first 4 months after emerging, 48% of the daily sap flow of freshly sprouted culms was provided by established culms via the rhizomes (Appendix Figure S6). It may well be that freshly sprouted culms not only receive water from the nearest directly connected established culm, but from several sources interconnected via the underground rhizome network. Generally, the dependency of freshly sprouted culms on established culms may vary with their distances to established culms; as such, a previous study on water transfer between poplar ramets found that ramets benefited more from proximal root connection than from distal ones (Adonsou et al., 2016). The current elongation stage of a given newly sprouted culm may further impact its dependency on established neighbors and thus the patterns and amounts of water it receives from donor culms.

Despite some previous indications of water transfer via rhizomes in bamboos (Dierick et al., 2010; Zhao et al., 2016), the mechanisms remain unclear. As such, the possible trade-offs between daytime sap flow of established culms and nighttime sap flow of freshly sprouted culms as well as the driving forces leading to the water transfer from the established donor culms to meet the water demand of the dependent freshly sprouted culms yet remain to be studied. Our results indicate that water supply from established donor culms to leafless freshly sprouted culms was reduced during the daytime, when the transpiration demand of established donor culms was high (more than 90% of whole-day transpiration; Figure 2, 3). In contrast, water transfer to leafless freshly sprouted culms was high during the nighttime, when transpiration of established culms is marginal. Such sap flow patterns indicate competition for water within bamboo clumps, particularly during the phenological period of bamboo sprouting.

According to the source-sink theory, resource translocation among connected herbaceous plants depends on resource availability; the resource is transferred from resource-abundant regions (source) to resource-scarce regions (sink; Marshall, 1996). The theory could explain the opposite water use patterns observed for freshly sprouted and established bamboo culms in our study. The established bamboo culms transpired a lot of water during the daytime, probably also retrieving water stored in the culms (Yang et al., 2015; Mei et al., 2016). During the nighttime, along with the largely reduced transpiration and the refilling of the culm water storage via root pressure mechanisms (Cao et al., 2012; Yang et al., 2015), water demand in established culms is largely reduced, while demand for water in freshly sprouted culms become relatively higher than in the established culms. Such resource allocation relies largely on resource availability of the donor individuals, which can be simulated with models (Caraco and Kelly, 1991) and which has previously been observed for several tree species (Fraser et al., 2006; Baret and DesRochers, 2011; Adonsou et al., 2016) and herbaceous species (Alpert and Mooney, 1986; Lau and Young, 1988; Chapman et al., 1992; Kroon et al., 1996; Stueffer et al., 1996; Wang Z. et al., 2011; Zhang et al., 2012). The dependency of freshly sprouted culms on established culms was largely relieved after they had fully developed leaves (Figure 2B,C). However, some water transfer among the young culms and the interconnected established culms continued for several weeks after leaf flushing. Substantial resource translocation could potentially be reactivated at a later time, e.g., in case of resource stress of individual culms (Marshall, 1996). For example, differences in soil water availability, which have been reported even for small patches, could be balanced via interconnected rhizome networks (Hutchings and Wijesinghe, 1997; Wang Y. et al., 2011; Zhang et al., 2012).

Another finding is that for the first 4 months after emergence, freshly sprouted culms kept active nighttime sap flow regardless of whether they were with or without leaves, contributing 69, 52, and 35% of total daily sap flow over the leafless, leafing and well-leaved periods, respectively (Figure 4). Reduced competitive water uptake from rhizomes by the established culms during the night could be a potential reason, while another could include carbohydrate translocation from the established culms to freshly sprouted culms during the night. It has been observed that non-structural carbohydrates in established culms were largely reduced during the period of sprouting of neighboring shoots, and it was assumed that they had been transferred into the freshly sprouted culms (Song et al., 2016). Carbohydrate transport from the source (usually the leaves) to the sink (e.g., rhizome or freshly sprouted culm) is believed to be driven by hydrostatic pressure gradients in the phloem (Münch, 1930). In the carbohydrate transport process, the phloem has to withdraw water from the surrounding tissues (usually the xylem), which usually equilibrates the water potential between the phloem and the surrounding tissues (Thompson and Holbrook, 2003; Hölttä et al., 2006). However, drawing water from the xylem is more difficult when the water potential in the xylem is more negative; carbohydrate transport in the phloem thus likely occurs during the nighttime, when xylem water potential is less negative (Hölttä et al., 2009; Savage et al., 2015). Without substantial transpiration demand and with the water storage refilling via root pressure mechanisms, the less negative water potential in established culms during the night further promotes the phloem to draw water and transport carbohydrates to freshly sprouted culms. However, the underlying mechanisms for water movement from established culms to freshly sprouted culms as well as its relationship with carbohydrate translocation remain unclear and will need to be addressed in future studies.

## Conclusion

In the period of sprouting, young bamboo culms receive water resources from neighboring established culms via the underground rhizome network. The freshly sprouted culms show a high share of nighttime water fluxes. This resource support may facilitate the very fast growth of the bamboo shoots, and enable the colonizing of new places.

## Data Availability

The datasets generated for this study are available on request to the corresponding author.

## Author Contributions

DF contributed to the experimental design, field installations, data analysis, and wrote and revised the manuscript. Particularly, DF conducted all the data analysis and figure-making, and wrote the preliminary draft of the manuscript. TM contributed to the experimental design, field installations, and revision for the manuscript. AR contributed to the field installations and revision for the manuscript. DH contributed to the experimental design and revision for the manuscript.

## Conflict of Interest Statement

The authors declare that the research was conducted in the absence of any commercial or financial relationships that could be construed as a potential conflict of interest.
