# Supplementary material for: Water Transfer Between Bamboo Culms in the Period of Sprouting
Source: Front Plant Sci. 2019 Jun 12;10:786. doi: 10.3389/fpls.2019.00786 (PMC6582707; doi:10.3389/fpls.2019.00786)
Supplement: Supplementary file 1 [file Data_Sheet_1.docx]

**Appendix**


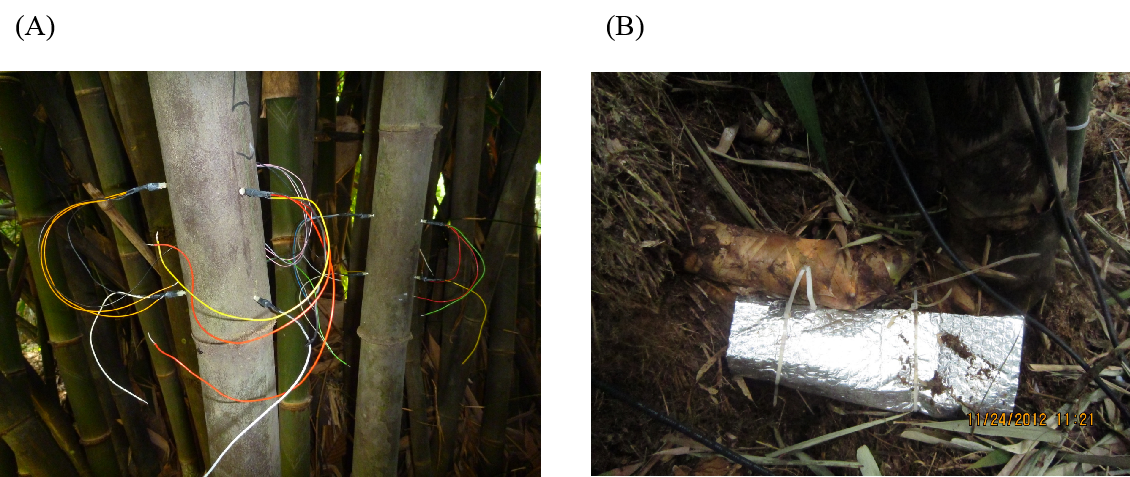


**Appendix Figure 1.** Field installation of TDP and the modified TDP for measuring sap flow of bamboo culms (A) and rhizomes (B), respectively.


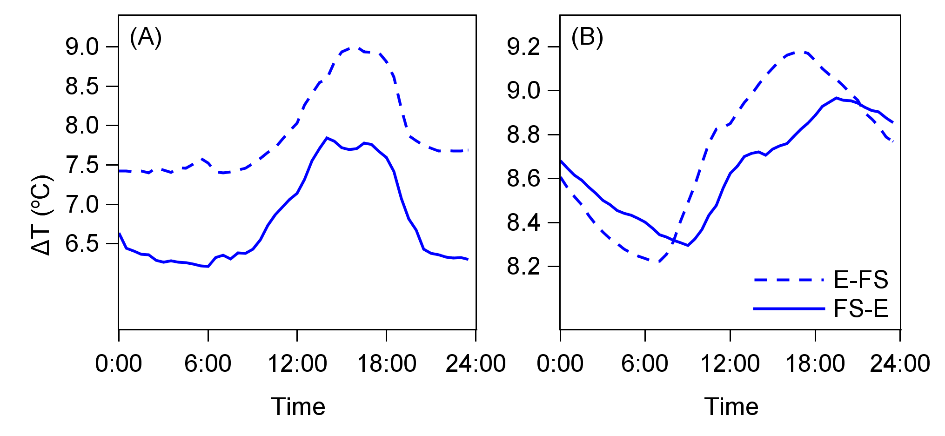


**Appendix Figure 2.** Typical diurnal patterns of temperature differences of upstream probes (ΔT_E-FS_) and downstream probes (ΔT_FS-E_) from rhizome: (A), temperature difference of downstream probes were lower than that of upstream probes (ΔT_E-FS_>ΔT_FS-E_); (B), temperature difference of downstream probes lagged behind that of upstream probes (ΔT_E-FS_ earlier than ΔT_FS-E_). Both scenarios implied flowing from established culm to freshly sprouted culm.


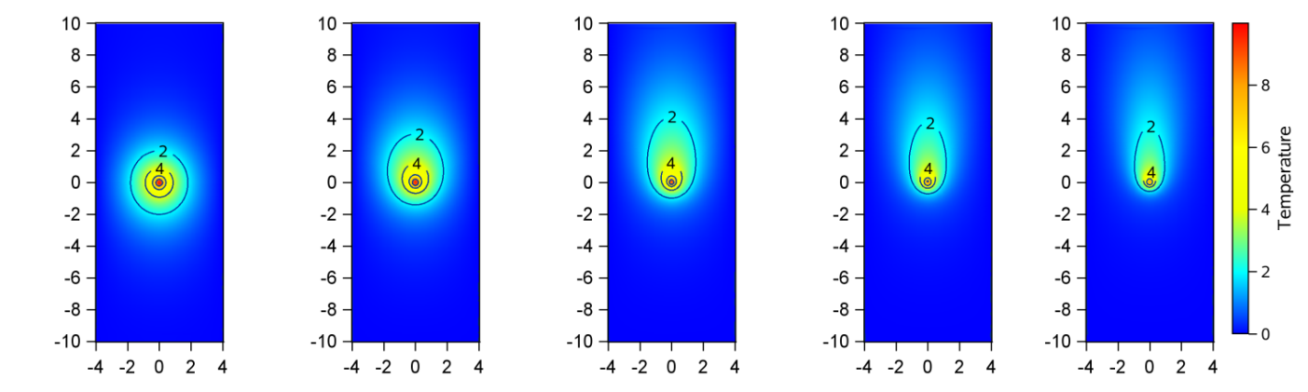


**Appendix Figure 3.** The simulated increased temperature fields (K) with different sap flux densities (from left to right: 0, 5, 10, 15, 20 g m^-2^ s^-1^). The values on x and y axis stand for the distances from the heating probe in tangential and axial directions, respectively.


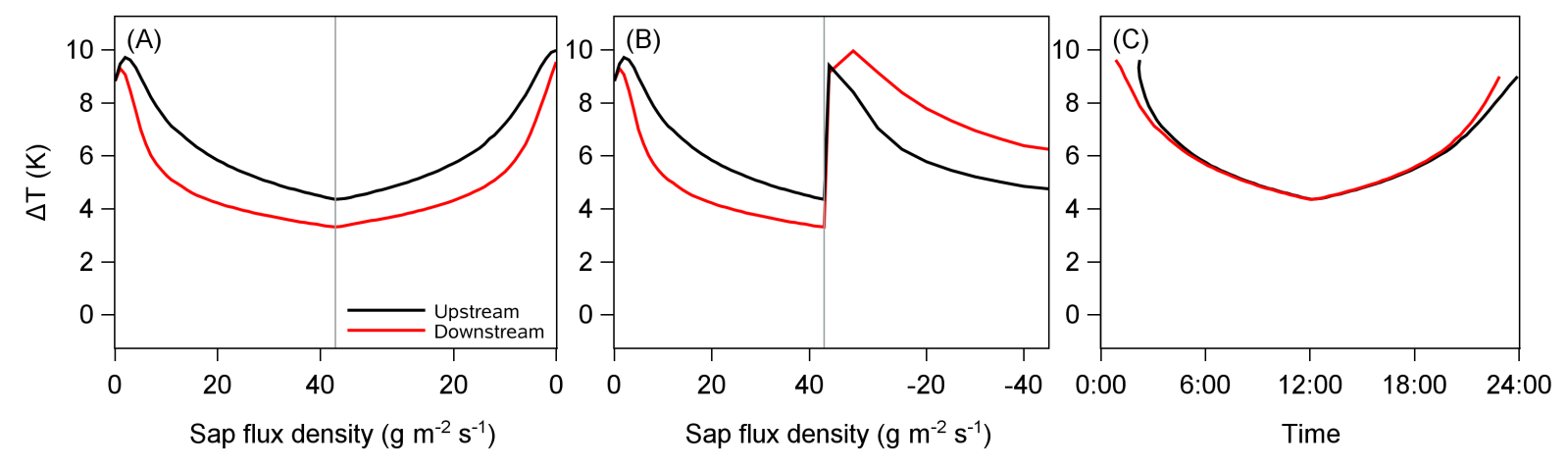


**Appendix Figure 4.** temperature difference (ΔT_up_ and ΔT_down_) with different sap flux densities (A) increasing from 0 to 43 g m^-2^ s^-1^ and then dropping down to 0 g m^-2^ s^-1^, (B) increasing from 0 to 43 g m^-2^ s^-1^ and then dropping suddenly down to 0 and increasing from 0 to 43 g m^-2^ s^-1^ in reverse direction, and (C) the proposed lagging patterns of temperature difference (ΔT_up_ andΔT_down_) in scenarios (3).


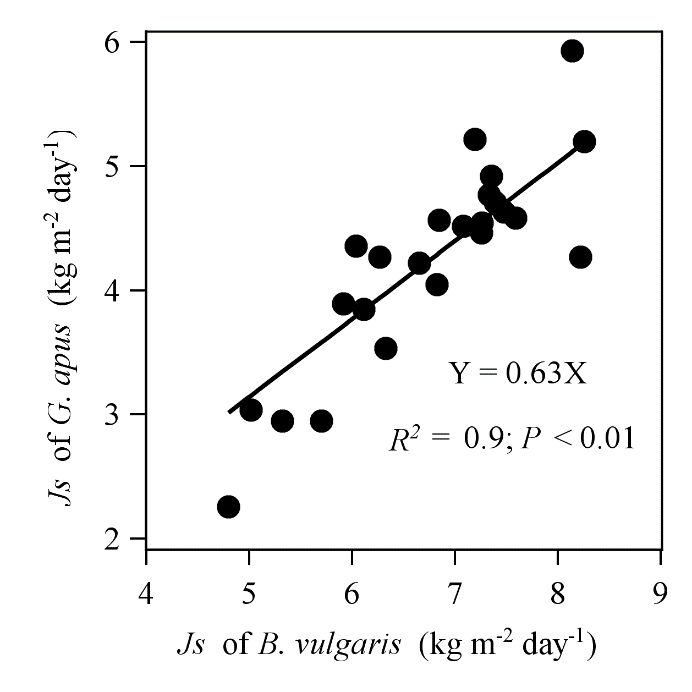


**Appendix Figure 5.** The linear relationship between *J_s_* (kg m^-2^ day^-1^) of established culms of *G. apus* and *B. vulgaris*. Data averaged from 5 culms of each species in 24 days ranging from Dec 29, 2012 to Feb 7, 2013.


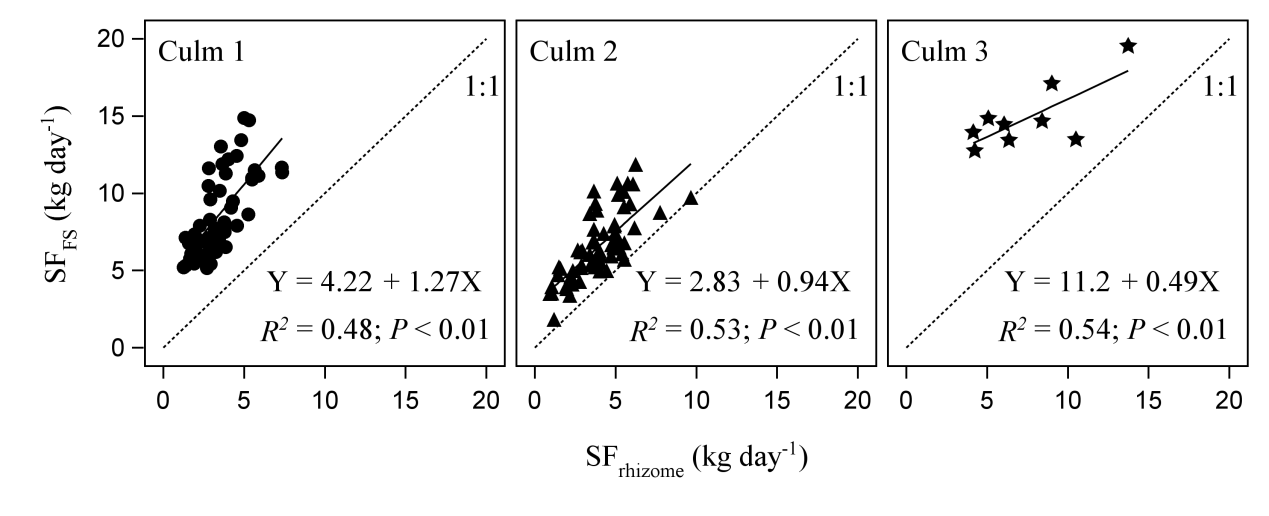


**Appendix Figure 6.** Estimated daily water use (kg day^-1^) of three freshly sprouted culms (SF_FS_) of *Bambusa vulgaris* in relation to water use of the corresponding rhizomes (SF_rhizome_). The water use of both freshly sprouted culms and rhizomes is based on the estimated sap flux density calculated with the adjusted formula for established culms, multiplied by the cross-sectional water conductive area at the location of the sensors.
